# Involving Health, Technology, and Financial Stakeholders in Co-Designing Digital Pathways for Value-Based Care

**DOI:** 10.2196/84885

**Published:** 2025-12-04

**Authors:** Pieter Vandekerckhove, Benjamin H L Harris, Louis J Koizia, Steven Howard

**Affiliations:** 1 Delft Centre for Entrepreneurship, Delft University of Technology, Jaffalaan 5, Delft, 2628 BX, The Netherlands, 31 15278980; 2 Erasmus School of Health Policy & Management, Erasmus University Rotterdam, Rotterdam, The Netherlands; 3 Department of Oncology, University of Oxford, Oxford, United Kingdom; 4 St. Catherine’s College, University of Oxford, Oxford, United Kingdom; 5 Department of Bioengineering, Cutrale Perioperative and Ageing Group, Imperial College London, London, United Kingdom; 6 Division of Medicine, Institute for Liver and Digestive Health, University College London, London, United Kingdom; 7 Department of Psychosocial Rehabilitation, Faculty of Health Sciences, Medical University of Lodz, Lodz, Poland; 8 Department of Health Services Administration, School of Health Professions, University of Alabama at Birmingham, Birmingham, AL, United States

**Keywords:** digital health, value-based health care, VBHC, patient-reported outcome measures, PROM, digital transformation, health care innovation, patient-centric care, health technology, patient-reported outcome, PRO, outcome measure, telehealth, telemedicine, eHealth, personalized, customized, engagement, patient-centered care, standardization, implementation

 Dear editor,

We read with great interest the recent viewpoint by Zhang et al [1] on leveraging digital technologies to integrate patient-reported experience measures and patient-reported outcome measures (PROMs) into value-based health care (VBHC). As researchers, practitioners, and educators committed to advancing VBHC, we share their enthusiasm and believe that digital tools have transformative potential.

However, this discussion is part of a longstanding struggle to operationalize VBHC in real-world practice. A fundamental shift is needed in how patient input is considered to both improve and innovate care delivery. Evidence suggests that survey-based approaches alone are insufficient; what is required is a more value-sensitive methodology that truly integrates the perspectives and needs of all stakeholders [2,3].

Such an approach demands horizontal and vertical engagement across the health system, embedding values through co-design processes. The example presented by Zhang et al illustrates this well: the collection of PROMs alone was not enough, but the oncology team’s subsequent actions to adapt care pathways led to more personalized care. Importantly, contrary to common assumptions, this did not result in greater complexity or reduced efficiency, but instead in more patient-centered care and higher levels of engagement.

The promise of digital tools will only be realized if challenges around digital access, health literacy, and inclusivity are addressed. Without careful attention, technology risks amplifying inequities rather than reducing them. Sustainable implementation also depends on overcoming barriers such as data interoperability, integration into clinical workflows, and building trust among clinicians and patients in the use of patient-reported data. Embedding patient-reported experience measures and PROMs into routine care further requires equipping health professionals with the skills to interpret and act on these measures, highlighting the central role of education and training in driving meaningful change.

In addition, the business models and revenue streams that underpin value-based transformations remain underexplored. Value-based care is only viable through sustainable financial reforms, which require adaptive strategies. Provider organizations’ legacy financial systems and processes are deeply rooted in fee-for-service payment, and it is difficult for them to transition to more advanced forms of alternative payment [4], supporting the need for incorporating strong financial incentives into VBHC and digital technology reforms. Previous studies have also demonstrated that diverse stakeholders are crucial for the design and implementation of sustainable VBHC business models [5]. Psychological and behavioral mechanisms underlying financial incentives may stand as barriers to VBHC and digital technology adoption. We therefore echo Zhang et al’s call for stakeholder involvement and argue that dynamic, participatory approaches to business model development are essential to achieving sustainable improvements in care quality and value.

In conclusion, digital technologies hold great promise in advancing VBHC, but realizing this potential requires more than technological adoption. It demands systemic reorientation toward value-sensitive design, stakeholder collaboration, equitable implementation, education and training of health professionals, and adaptive business modeling. We encourage future research that not only evaluates outcomes but also tests models for scaling and sustaining these approaches across diverse health systems.

## Abbreviations

PROM: patient-reported outcome measure
VBHC: value-based health care
